# Multiplexed laser particles for spatially resolved single-cell analysis

**DOI:** 10.1038/s41377-019-0183-5

**Published:** 2019-08-21

**Authors:** Sheldon J. J. Kwok, Nicola Martino, Paul H. Dannenberg, Seok-Hyun Yun

**Affiliations:** 1 Harvard Medical School and Wellman Center for Photomedicine, Massachusetts General Hospital, 50 Blossom Street, Boston, MA 02114 USA; 2 Harvard-MIT Health Sciences and Technology, Massachusetts Institute of Technology, 77 Massachusetts Avenue, Cambridge, MA 02139 USA; 3 LASE Innovation Inc., 650 E. Kendall Street, 2nd FL, Cambridge, MA 02142 USA

**Keywords:** Biophotonics, Microresonators, Imaging and sensing

## Abstract

Biomolecular analysis at the single-cell level is increasingly important in the study of cellular heterogeneity and its consequences, particularly in organismic development and complex diseases such as cancer. Single-cell molecular analyses have led to the identification of new cell types^1^ and the discovery of novel targets for diagnosis and therapy^2^. While these analyses are performed predominantly on dissociated single cells, emerging techniques seek understanding of cellular state, cellular function and cell–cell interactions within the native tissue environment by combining optical microscopy and single-cell molecular analyses. These techniques include in situ multiplexed imaging of fluorescently labeled proteins and nucleotides, as well as low-throughput ex vivo methods in which specific cells are isolated for downstream molecular analyses. However, these methods are limited in either the number and type of molecular species they can identify or the number of cells that can be analyzed. High-throughput methods are needed for comprehensive profiling of many cells (>1000) to detect rare cell types, discriminate relevant biomarkers from intrinsic population noise, and reduce the time and cost of measurement. Many established, high-throughput single-cell analyses are not directly applicable because they require tissue dissociation, leading to a loss of spatial information^3^. No current methods exist that can seamlessly connect spatial mapping to single-cell techniques. In this Perspective, we review current methods for spatially resolved single-cell analysis and discuss the prospect of novel multiplexed imaging probes, called laser particles, which allow individual cells to be tagged in tissue and analyzed subsequently using high-throughput, comprehensive single-cell techniques.

## Single-cell analysis: Laser particles help break the color barrier

Techniques that connect spatial information about cells, such as morphology or migration behavior, with high-level genetic testing can be sped up using imaging probes that emit hundreds of distinct colors. Seok-Hyun Yun from the Massachusetts General Hospital in the United States and co-workers review efforts to tag cells with light-emitting labels to track them with optical microscopes and across different analytical platforms. Most approaches, such fluorescent antibody-based detection, only offer a few colors for cell tracking. Recent results with so-called “laser particles”, tiny disks made from gallium arsenide-type materials, indicate that these probes offer significantly improved throughput. Experiments show that silica-encased laser particles can be readily internalized into a variety of cell types and emit over 400 distinct colors, enabling cell tracking even in dense, scattering tissue.

## Current methods for spatially resolved single-cell analysis

**Fig. 1:**
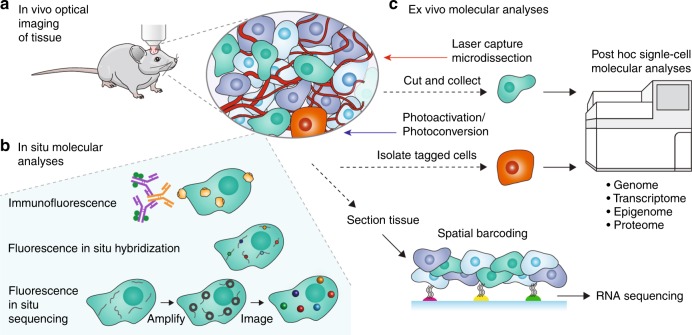
Overview of methods for spatially resolved molecular analysis. **a** Imaging in tissues provides information on where cells are located with respect to each other and their environment, as well as observation of dynamic behaviors in their native environment. **b** Biomolecules can be imaged in situ with high-resolution fluorescence imaging but with limited multiplexing. Proteins can be imaged with immunofluorescence. Fluorescence in situ hybridization enables imaging of targeted nucleic acids, including DNA and RNA. Fluorescence in situ sequencing enables imaging of untargeted transcriptomic mRNA expression. **c** Laser microdissection and photoactivation/photoconversion enable the selection of specific cells of interest for downstream molecular analyses. Spatial barcoding of tissue sections enables high-throughput spatial transcriptomics, but not at single-cell resolution

Optical fluorescence microscopy has been a powerful tool to understand cellular function and phenotypic heterogeneity (Fig. 1a). Image-derived phenotypes comprise spatial information such as morphology and location relative to other cells, as well as dynamic behaviors, such as migration and invasion, and result from the complex interplay of different cell types within tissues. Fluorescence imaging at high spatial resolution can also provide molecular analysis in situ within each individual cell (Fig. 1b), thereby enabling correlation of image-derived phenotypes and molecular profiles. The most pervasive approach is the antibody-based fluorescence detection of specific proteins after tissue fixation and permeabilization. Repeated antibody elution and staining steps^4^ or the use of DNA-barcoded antibodies^5^ can extend multiplexed detection from 4~40 proteins per sample. Another approach involves imaging RNA in cells with single-molecule fluorescence in situ hybridization (smFISH) using targeted oligonucleotide probes. For multiplexed detection, sequential rounds of smFISH imaging can detect ~100 distinct RNA species in single cells^6^. Enzymatic amplification of RNA species to generate cDNA nanoballs (amplicons) in fluorescence in situ sequencing (FISSEQ) enables orders of magnitude higher signal intensity compared to that of smFISH^7^. An approach called STARmap combines RNA amplification, tissue optical clearing and fluorescence*-*based readout to detect up to ~1000 genes in brain tissue at single-cell resolution^8^.

Although in situ molecular profiling techniques retain spatial information, their scope is limited compared to that of widespread single-cell technologies that analyze dissociated suspensions of cells devoid of spatial information. Current multiplexed in situ techniques primarily measure RNA, do not cover the whole transcriptome (>10,000 expressed genes), and require laborious imaging steps. In contrast, single-cell technologies based on next-generation sequencing (NGS) techniques comprise a suite of “omic” methods that can more comprehensively profile the genome, epigenome, transcriptome and proteome^3^. These methods include more established techniques, such as high-throughput single-cell DNA and RNA sequencing (scDNA-seq and scRNA-seq), to capture the whole genome and transcriptome, respectively, as well as single-cell mass cytometry for multiplexed analysis of proteins^9^. Emerging epigenetic techniques that probe how gene expression is regulated include ChIP-seq (chromatin immunoprecipitation and sequencing) and ATAC-seq (assay for transposase-accessible chromatin using sequencing). However, as mentioned, all of these technologies require tissue dissociation into cellular suspensions, which leads to the loss of all spatial information.

Several techniques restrict molecular analysis to specific cells that can be defined and selected during imaging (Fig. 1c), thereby retaining spatial information, but with a tremendous cost to throughput. Methods based on photoactivation or photoconversion^10^ involve tagging of selected cells while imaging and subsequent isolation of the selected cells or cellular material for downstream analyses. Laser capture microdissection (LCM) is a more mature technology that involves the isolation of cells from tissue by laser ablation under the microscope. LCM has been employed in an automated manner to couple with single-cell genomic^11^, transcriptomic^12^ and proteomic profiling^13^ to obtain spatially resolved molecular profiles. Instead of isolating individual cells for analysis, significantly higher throughput can be achieved with spatial barcoding. One method uses an array of DNA-barcoded oligonucleotides on glass slides that capture mRNA from permeabilized tissue sections^14^. Subsequent RNA-seq is mapped to the spatial coordinates of the array and thus the tissue. While this approach improves throughput compared to LCM, it is limited to RNA profiling and does not maintain depth information about cells within the tissue slice. At present, there is no high-throughput method that permits comprehensive molecular analysis with the full complement of single-cell omics technologies while retaining spatial information.

## Current methods for multiplexed cell tagging

Cell tagging enables the tracking of cellular identities over time and space and potentially across analytical platforms to correlate spatial and molecular analysis. However, conventional fluorescent probes allow only a few different cell types to be labeled with different colors due to spectral overlap. Recently, the development of multiplexed imaging techniques has permitted the labeling and tracking of a far greater number of cellular populations, a new paradigm for biological imaging, which has gained traction given the increasing appreciation of intercellular diversity. Tracking individual cells through imaging gives access to their properties (e.g., spatial location, morphology) over time, as well as the spatial relationships between different cells. The combinatorial expression of multiple fluorescent proteins in each cell can achieve up to ~100 resolvable colors using ratiometric coding^15^. This technique has been used to discriminate neurons in brain tissue^15^ and for long-term cell tracking in zebrafish skin^16^. Quantum dots have also been employed for image-based multiplexing in vitro^17^. This ratiometric or image-based coding, however, is subject to error for thick tissues due to variable attenuation and scattering. Other approaches proposed to overcome this limitation include multiplexed lifetime imaging with lanthanide nanoparticles^18^ and Raman multiplexed probes with 24 colors^19^. However, none of these techniques are easily scalable to a much larger number (>1000) of cells.

## Laser particles for single-cell analysis

A new class of nano/microsized imaging probes has emerged that harness narrowband laser emission to enable massively multiplexed cell tagging. These laser particles (LPs) are particularly promising for single-cell analysis for several reasons. First, they provide a means for narrowband laser emission from inside cells, This enables cell tagging by spectral barcoding, which is far more reliable than barcoding based on intensity levels or imaging features that are of limited use in tissue due to wavelength-dependent absorption and scattering. Second, these spectral barcodes can be repeatedly read in a nondestructive manner. This allows cell tracking over time and space in biological systems, as well as the retention of individual cellular identity across different analytical platforms, which enables the association of spatial information and other image-derived phenotypes with molecular profiles.

**Fig. 2:**
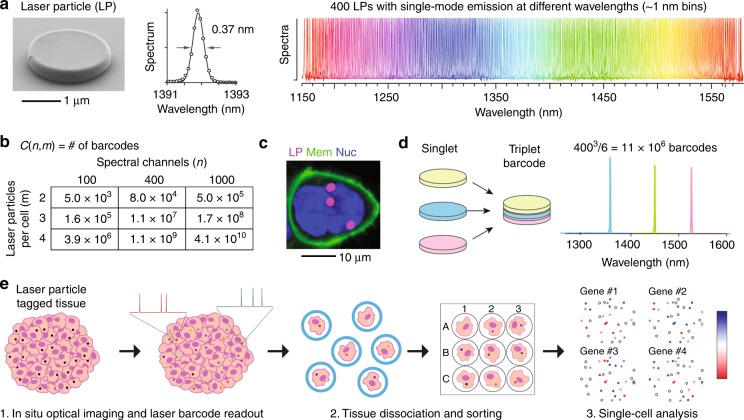
Laser particles for comprehensive single-cell analyses. **a** Left: SEM of a silica-coated, III–V semiconductor microdisk laser particle (LP). Middle: Narrowband emission measured from the LP upon optical pumping with a 1060 nm laser emitting 3 ns pulses at 2 MHz and 20 pJ/pulse. Right: Use of different materials and fabrication of different microdisk diameters by nanolithography yields 400 LPs with single-mode emission from 1170 to 1580 nm. Reproduced from Ref 25. **b** Spectral barcoding by using combinations of different LPs per cell. **c** A human cell (HEK293) carrying three LPs. Magenta (LP), green (membrane), blue (nucleus). **d** Left: Fabrication of multiplet LPs. Right: An example of emission from a triplet LP, one of ~11 million barcodes possible assuming 400 spectral channels. **e** Proposed workflow for comprehensive single-cell analysis using LPs. Tissue-containing laser particles are imaged to capture tissue architecture and cellular behaviors. LP emission is measured before and after dissociation to track cell identity. Any downstream molecular analysis of single cells (e.g., transcriptomics and proteomics) can be correlated to their location and behavior in their native tissue

Several potential designs have been reported, including fluorescent polystyrene particles^20,21^, upconverting nanoparticle microlasers^22^, perovskite nanowires^23^, and plasmonic nanoparticles^24^. Our recent study employed silica-coated III–V semiconductor microdisk LPs with diameters of ~2 μm and total thicknesses of ~400 nm^25^. Our microdisk LPs have several key properties that make them more suitable for cellular barcoding than the previous designs, including relatively small sizes (~0.1% of the cell volume), stability in aqueous environments, biocompatibility, and tunable wavelengths over a wide range. Silica-coated microdisk LPs were readily internalized into a variety of cell types through macropinocytosis and had no appreciable effect on cell viability, cell cycle time, or motility when internalized inside cells^25^. Using five different semiconductor alloy compositions and stochastically varying the microdisk diameter through nanofabrication enabled single-mode emission over a wide range in the tissue-penetrating NIR-II window, from 1170 to 1580 nm, with subnanometer linewidths (Fig. 2a). Given 1-nm bins within which LP emission is stable, this range provides more than 400 unique colors, with room for further expansion using different materials (e.g., InGaAsP and InAlGaAs for NIR-I and NIR-II, InGaN for visible). The use of the NIR range also preserves compatibility with conventional fluorescent probes in the visible wavelengths that can label different cellular features (e.g., nuclei, membrane). The availability of hundreds of spectrally distinct colors for multiplexed imaging enables longitudinal cell tracking even in dense, scattering tissues. In our study^25^, we used microdisk LPs to track thousands of individual cancer cells over several days in a 3D tumor spheroid invasion model. Our data enabled the classification of individual cells according to their motility and the identification of cells moving together in small packs within the spheroid by analyzing spatial correlations in velocities.

Despite progress in the development of multiplexed imaging probes, the number of resolvable colors, or available barcodes, is still far fewer than desired for most applications (~10,000 cells per 1 mm^3^ of tissue volume). To tag cells uniquely, the number of barcodes should exceed the number of cells. Spectral barcoding with LPs is a practical and scalable approach to break through this limitation. LPs with different emissions can be combined to readily achieve millions of unique barcodes (Fig. 2b). One approach is the uptake of multiple LPs in each cell (Fig. 2c), as long as LPs can be colocalized to specific cells by segmentation of cell boundaries. However, this approach would not be suitable for dividing cells since barcode fidelity would be lost upon LP segregation. A more tractable approach is using multiple LPs stacked together in a single particle to generate an emission composed of multiple laser lines^25^ (Fig. 2d). To associate spatial information with single-cell analyses, the emission spectra of LP tagged cells can be read during imaging to catalog the position and identity of cells within tissue and following tissue dissociation at an appropriate step before subsequent analyses (Fig. 2e). For plate-based sequencing, LP emission can be measured during or after the sorting of single cells into individual microwells. LP cell tagging is also compatible with high-throughput methods, including microdroplet-based^26^ and split-pool barcoding^27^, which introduce cell-specific DNA barcodes for highly parallel analysis as long as an association between the optical LP barcode and the DNA barcode is made.

Further development of the LP technology could enable the phenotypic and molecular characterization of every cell in a tissue, limited only by light penetration during imaging. One critical area of research is cancer biology. A major question is how, when, and which cancer cells within a heterogeneous tumor become metastatic. Live imaging and multiplexed cell tracking with LPs in dense tumor tissue can delineate key steps in the metastatic cascade, including migration, intravasation, and extravasation, as well as interactions with the tumor microenvironment. Linking these cellular phenotypes with molecular profiles could address the largely unexplored roles of cellular cooperation and non-cell-autonomous behaviors at the single-cell level. Analysis of interacting ligands and receptors on individual cells may lead to the construction of a comprehensive, spatially resolved cell–cell interaction network to reveal functional mechanisms in metastatic tumor progression. Beyond research applications, similar experiments conducted on patient-derived tumor models could identify biomarkers for diagnosis or targets for therapy.

Another application of single-cell analyses, particularly scRNA-seq, is unbiased cell classification, which has been successful in identifying new cell types in a number of different tissues. One reason for their success is a data-driven approach that is unencumbered by previous biases towards cellular identity. Techniques that integrate multiple omics layers of data (e.g., transcriptomic and epigenomic) provide a more complete picture of a cell’s molecular framework that is thus more powerful than using any single layer alone^3^. Cellular phenotypes captured by imaging can be considered additional layers of information that reflect a cell’s native environment. Since cells do not naturally exist in isolation, this information is essential for understanding cellular state, function, and cell–cell interactions in their native tissue environment. A recent initiative by an international consortium to characterize every cell in the human body, the Human Cell Atlas, has highlighted the need for spatial information to generate comprehensive reference maps as a basis for diagnosing, monitoring, and treating disease^28^. Spatially resolved single-cell analysis will likely transform applications such as tissue engineering and precision medicine. Comprehensive datasets combining imaging and molecular profiles could also be used to train machine-learning algorithms for image-based cell classification and in silico diagnostics.

In conclusion, advances in cellular imaging and molecular analyses have pushed the envelope of what can be observed and measured, but their impact has been hampered by the inability to employ these technologies in tandem on the same cells. Laser particles are a novel class of multiplexed imaging probes with the potential to bridge these technologies by enabling massively multiplexed imaging, cell tagging, and comprehensive single-cell analysis.
